# Variation of growth characteristics of pneumococcus with environmental conditions

**DOI:** 10.1186/s12866-019-1671-8

**Published:** 2019-12-26

**Authors:** Adrienn Tóthpál, Katherine Desobry, Shreyas S. Joshi, Anne L. Wyllie, Daniel M. Weinberger

**Affiliations:** 1 Epidemiology of Microbial Diseases, Yale School of Public Health, 60 College Street, New Haven, CT 06510 USA; 2 Institute of Medical Microbiology, Semmelweis University, Nagyvarad ter 4, Budapest, HU-1089 Hungary

**Keywords:** Pneumococcal infection, Growth curves, Temperature, Carriage, Anaerobic growth

## Abstract

**Background:**

Pneumococcus is exposed to a variety of temperature and oxygen levels in the upper respiratory tract and as it invades the lung, tissues, and blood. We sought to determine the effect of environmental variability on growth in vitro and to assess variability between strains. We evaluated the effect of temperature and oxygen on the growth of 256 isolates representing 53 serotypes, recovered from healthy carriers and disease patients. Strains were grown at a range of temperatures, anaerobically or in ambient air with catalase, and were monitored by reading the optical density. Regression models evaluated variation in the characteristics of the growth curves.

**Results:**

Most isolates grew to the maximal density at low temperatures (~33C) and under aerobic conditions. There was considerable variability between strains, and some of this variability was linked to serotype. However, capsule-switch experiments suggest that the production of different capsules might not be sufficient to explain this variation, suggesting there could be interactions between the capsule and genetic background.

**Conclusions:**

Pneumococcal strains vary in how they respond to environmental variations, some of this variation can be explained by the capsule type being produced, but capsule production itself is not sufficient to explain the variability. This variability could help to explain why different lineages of pneumococcus are more common in carriage or disease.

## Background

*Streptococcus pneumoniae* (pneumococcus) is an opportunistic pathogen that resides in the human nasopharynx. The nasopharynx is considered to be the reservoir of transmission between individuals [1]. Pneumococci are diverse, with > 90 serotypes (defined by the capsule polysaccharide) and has tremendous genetic variation, resulting from recombination. Serotypes vary in their prevalence among healthy carriers and in the likelihood that they will cause severe disease [2]. As conjugate vaccines against 7, 10, and 13 pneumococcal serotypes have been introduced, the vaccine-targeted serotypes have declined in frequency among healthy carriers and as causes of disease, while serotypes not targeted by the vaccine have increased in importance (serotype replacement). Next-generation conjugate vaccines are under development that target additional serotypes, and it is likely that these vaccines will lead to further serotype replacement. Understanding the factors that influence the fitness of these non-vaccine strains could help to anticipate future patterns of serotypes replacement and could aid in the design of more optimal vaccines.

The success of pneumococcus in the nasopharynx and the likelihood that it causes disease is likely driven, in part, by how it responds to variations in its local environment. In different anatomical sites within the human host, pneumococci are exposed to variable temperature and oxygen levels. In the nasopharynx, considered its main niche, the average temperature is around 33 °C, with some differences between children and adults [3–6]. The core body temperature, which would be encountered during invasion into tissues, is 37 °C. The temperature in the lungs is constantly changing based on the temperature of inhaled air but is generally lower than 37 °C [7]. During infection by pneumococci or during viral co-infection (such as influenza or respiratory syncytial virus), both external and internal temperature increases [8–12]. Oxygen levels also vary within the host. In the nasopharynx, bacteria on top of the mucus layer are exposed to almost ambient air (20% O_2_). Pneumococci in biofilms in the nasopharynx encounter lower levels of oxygen [13]. Entering the lower respiratory tract or the middle ear, pneumococci are exposed to micro-aerophilic conditions and to almost anaerobic conditions when present in blood and the cerebrospinal fluid (CSF) [14–17]. Likewise, mucus production during infection (i.e., due to viral infection) can block the air passage and form micro-aerophilic (around 5% O_2_) or even anaerobic microenvironments [15, 16].

While pneumococci exist in a complex environment, simple in vitro experiments can be used to isolate the response to specific environmental conditions. Laboratory studies have evaluated variations in growth characteristics by serotype and have identified a relationship between in vitro growth characteristics and prevalence of serotypes in the nasopharynx of healthy children [18, 19]. However, the effect of variation in temperature and oxygen on the growth of different strains and serotypes has not been systematically explored.

The aim of this study was to investigate how environmental variability in temperature and oxygen influences the growth characteristics of pneumococci and how the responses to these varies by strain. Using a diverse set of clinical and nasopharyngeal isolates, as well as capsule-switch and capsule-knockout variants generated in the lab, we quantified how the growth characteristics of pneumococci in vitro vary under a range of temperatures and in aerobic and anaerobic conditions. Using statistical models, we estimated the variation in these growth characteristics associated with serotype (after adjusting for isolate-specific effects that could reflect culture history or other characteristics).

## Results

### Descriptive results

**Fig. 1:**
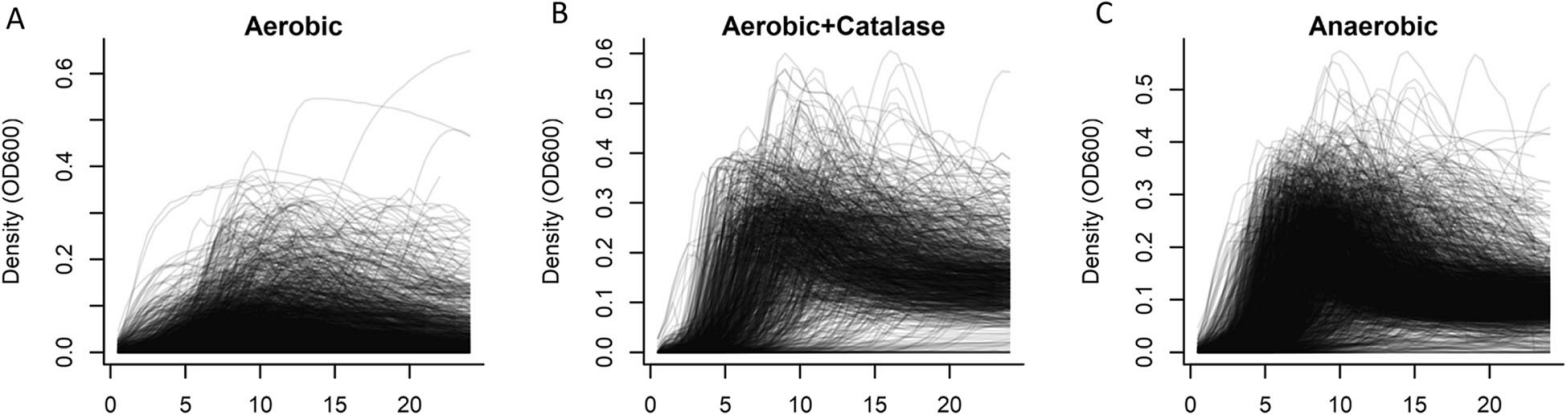
Variation in growth curves among all isolates used in this study. Growth was measured every 30 min over 24 h at 30–39 **°**C. (**A**) Ambient air, (**B**) ambient air with catalase, and (**C**) anaerobic conditions. Each line corresponds to an individual growth curve

In total, we performed more than 4900 growth curves on 256 different pneumococcal strains, representing 53 different serotypes **(**Fig. 1**)**. Because of the relatively poor growth achieved in aerobic conditions without catalase, all further analyses presented here focus on the results from clinical samples grown either with catalase or anaerobically (*N* = 3151). There was a weak, positive correlation (rho = 0.27, 95%CI: 0.24, 0.30) between the maximum density achieved and maximum growth rate and no notable correlation between the length of the lag phase and either growth rate (− 0.06; 95%CI: − 0.09, − 0.02) or maximum density (0.04; 95%CI: 0.00, 0.07). Growth curves for all isolates and conditions can be explored interactively at https://weinbergerlab.shinyapps.io/ShinyGrowth_v2/.

**Fig. 2:**
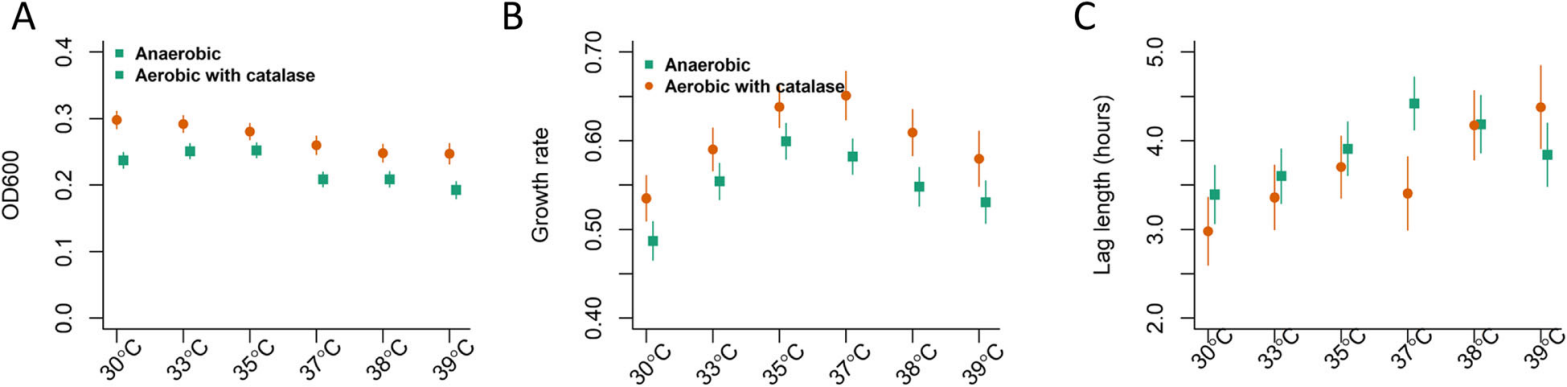
Effect of temperature and oxygen on growth phenotypes. (**A**) Maximum density achieved, (**B**) Maximum growth rate and (**C**) length of lag phase. Anaerobic (green squares) and aerobic+catalase (orange circles). These estimates are based on 3151 growth curves. Mean+/− 95% confidence intervals, calculated from a regression model adjusting for serotypes, presence of oxygen, temperature, site of isolation, and an interaction between temperature and presence of oxygen

The maximum density of the strains was similar at 30–35 °C, and lower densities were achieved at 37–39 °C (Fig. 2a); maximum density was greater in aerobic conditions (with catalase) than in anaerobic conditions. The growth rates were fastest at 35–37 °C, with slower growth at lower or higher temperatures (Fig. 2b); growth was faster in aerobic conditions (with catalase) than anaerobic conditions. The length of the lag phase increased with temperature (Fig. 2c).

### Variation in growth characteristics associated with serotype

**Fig. 3:**
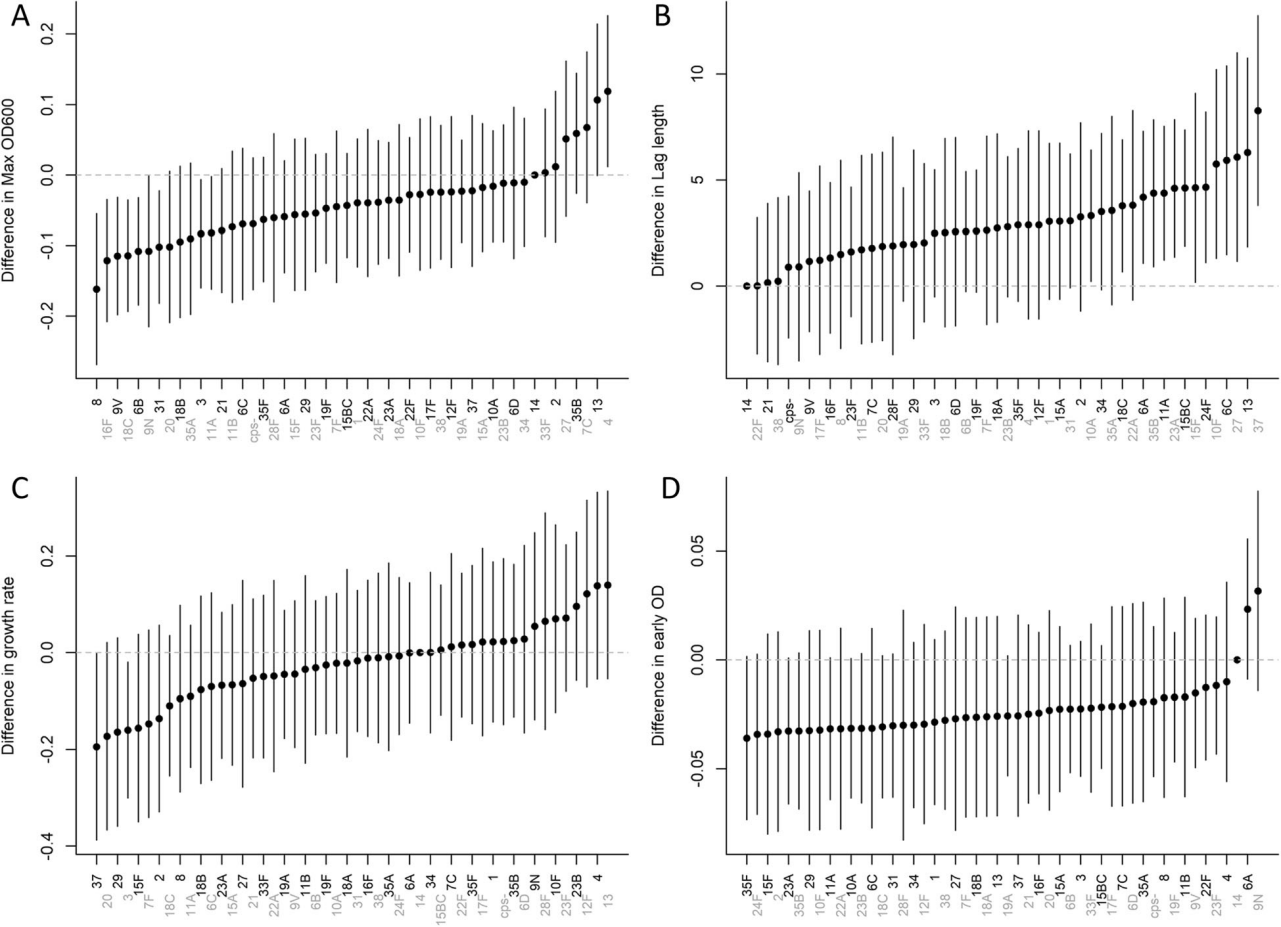
Variation in growth characteristics associated with serotype. Variation in (**A**) maximum density (**B**) length of the lag phase (**C**) maximum growth rates and (**D**) density at an early time point. The dots represent regression coefficients +/− 95% confidence intervals, as calculated from a regression model that controls for presence of oxygen, temperature, site of isolation, and interactions among these. The reference in the regressions is serotype 14

We quantified variation in maximum density achieved, growth rate, and length of lag phase associated with serotype. A number of serotypes differed from the reference (serotype 14) in maximum density achieved, length of the lag phase, and the density at an early time point (Fig. 3). For instance, the clinically-relevant serotypes 3, 6B 8, 9 V, 11A, 16F, 18C and 31 grew to a lower maximum density on average than serotype 14.

We next sought to determine whether this variability in growth characteristics was due to serotype or due to other genetic variability. Some of the serotypes in our collection were represented by multiple genetic lineages (MLST types). There did appear to be variation in growth characteristics associated with serotype that was similar across multiple isolates and MLST lineages (Additional files 1, 2: Figures S1, S2). However, there was not enough diversity in our sample to do a formal analysis. We therefore evaluated the growth characteristics of capsule-knockout strains as well as several capsule-switch variants of the reference strain TIGR4. While the results were ambiguous, they suggested that there was an effect of capsule production on maximum density, and this effect was more pronounced during anaerobic growth compared with aerobic growth with catalase (Additional files 3-6: Figures S3-S6). However, there was little correspondence in the serotype-specific patterns observed with the capsule-switch variants and the clinical isolates, suggesting the genetic background could play a role.

### Effect of oxygen on growth varies by serotype

**Fig. 4:**
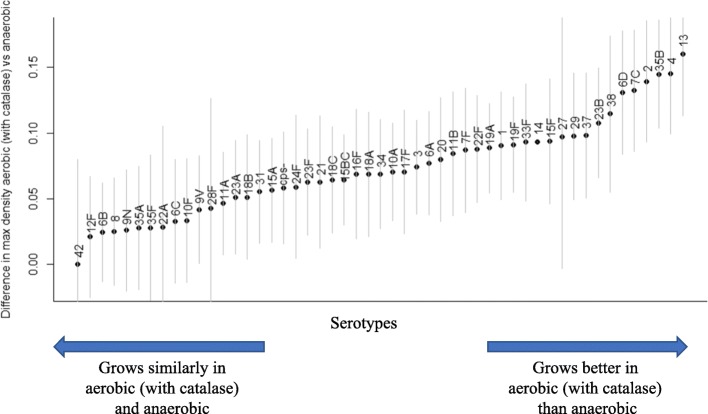
Difference in maximum density by serotypes when grown in aerobic conditions with catalase versus anaerobic conditions. Positive values indicate that a higher density (as measured by optical density) was achieved under aerobic conditions. A value of 0 indicates no difference. Mean+/− 95% confidence interval, as calculated using a regression model with covariates for serotype, presence of oxygen, temperature, an interaction between serotype and presence of oxygen, and a random intercept for the isolate. The reference in the regression is serotype 14

We considered whether the effect of oxygen on growth patterns varied by serotype (Fig. 4). Overall, isolates producing certain capsules (e.g., 2, 4, 13, 35B) grew to a higher density under aerobic conditions (with catalase), while isolates producing other capsules (e.g., 6B, 8, 9 N, 12F) did not show a difference between aerobic and anaerobic growth (Fig. 4). This pattern was similar for maximum growth rate. For lag phase, there was little difference among the serotypes in how they responded to the presence of oxygen, with only the serotype 6C and 13 isolates exhibiting a longer lag phase in aerobic conditions.

## Discussion

We provide novel information about how the response to environmental conditions can differ between pneumococcal isolates. Testing a diverse set of strains, pneumococci grew to the highest density under conditions that mimic the normal environment of the nasopharynx in terms of temperature and oxygen level. Important differences in these patterns were observed between isolates. While, some of this variability could be due to the culturing history of the individual isolates, patterns of variation were apparent by serotype, and these patterns were consistent across multiple MLST lineages. However, experiments with capsule-switch strains did not yield clear results, suggesting a possible role for and interaction with the genetic background.

When comparing growth in aerobic conditions (with catalase) with growth in anaerobic conditions, the benefit of oxygen varied by serotype (Fig. 4). Serotypes 2, 4, 13, 23B, 35B and 38 grew better with additional catalase than in anaerobic conditions, whereas serotypes 6B, 8, 9 N, 12F, 22F and 42 grew similarly in both environments. We tested a relatively small number of isolates per serotype, so it is possible that non-capsular genetic variations or differences in the culture history of the isolates could influence the observed responses to oxygen.

As the nasopharynx is the normal habitat of pneumococcus, we had hypothesized that pneumococci would grow optimally at temperatures in the low-30°C range, similar to the temperature of the nasopharynx. Indeed, temperature played an important role in terms of the maximal density achieved and how quickly the isolates started growing (lag phase). Isolates grew to the highest density and had the shortest lag at temperatures resembling those of the nasopharynx; these patterns were particularly pronounced for those isolates obtained from the nasopharynx. These patterns among the nasopharyngeal isolates are unlikely to be a result of the in vitro culture history of the strains alone—pneumococcal isolates are typically cultivated at 37 °C, and thus if the patterns were a result of adaptation, we would expect more efficient growth at that temperature rather than at 33 °C. These findings, along with recent work on the effect of lower temperatures on the immune response to pathogens in the upper respiratory tract [20], suggest that the environment of the nasopharynx is optimal for pneumococcal growth.

During the invasion process, when the pneumococcus transitions from the nasopharyngeal environment to the internal body environment, it has to adapt to many changes, including nutrient, temperature and oxygen levels. Temperatures vary from the low-30 °C range in the upper respiratory tract to 37 °C in core body sites and even higher during fever. Likewise, oxygen levels can vary considerably during infection. Increased mucus production (due to co-infection with viruses or other pathogens in the upper respiratory tract) leads to lower oxygen levels and may generate a local hypoxic environment [21–23]. The availability of oxygen is also decreased in pneumonia, empyema, and otitis media. Oxygen levels in the uninflamed middle ear space, for example, resemble that of venous blood, are less than a third that of the airway, and may be further reduced by the presence of effusion [14, 15, 24]. Some isolates appeared to be strongly influenced by these variations in oxygen and temperature, indicating that the strains have the capability of adapting (either in the host or during in vitro growth) to thrive under different conditions.

Some of the differences observed in growth phenotypes between carriage and disease isolates could reflect opaque/transparent phase variation [25]. Opaque variants are generally isolated from IPD and have increased capsule production and decreased production of certain surface proteins. Phenotypically, the presence of oxygen accentuates differences in capsule production between opaque and transparent variants [24]. This effect could be mediated via the pathways involved in converting pyruvate to acetyl-CoA, an important biochemical precursor for capsule production for many serotypes [26]. Variations in the use of this pathway between serotypes or the efficiency of this pathway between lineages could influence some of the patterns that were observed in the growth curves.

This study had certain limitations. For the growth curves, we used BHI broth which is an artificial growth medium that differs in nutrient composition from the host. We evaluated several minimal media but found that growth was generally poor, making comparisons between strains difficult. While we tested a large number of strains representing many serotypes, some serotypes were only represented by a single isolate (i.e., 11B, 12F, 13). This could limit the generalizability of serotype-specific findings in these instances, making it difficult to make inferences about whether variability was due to serotype, site of isolation, or lineage effects. The strains used in this study were largely a convenience sample from clinical studies. The genetic diversity of pneumococcus makes it difficult to draw conclusions about the cause of differences between strains. The growth curves with the capsule-knockout strains and capsule-switch variants suggests that the capsule itself could influence these phenotypes. We did not perform any gene expression studies, which could be highly influenced by environmental conditions [27]. Further work could explore the genetic basis (both capsular and non-capsular factors) for the differences in growth phenotypes between strains.

In conclusion, we demonstrate that the growth characteristics of pneumococcus are influenced by environmental variations, that the effect of these variations depend on strains, and that the optimal growth conditions for carriage isolates resemble the conditions of the nasopharynx.

## Methods

### Bacterial strains, culture media, and chemicals

#### Strains

Invasive pneumococcal disease (IPD) isolates were obtained from the isolate bank at the Centers for Disease Control/Active Bacterial Core surveillance; carriage isolates were provided by Ron Dagan (Ben-Gurion University, Israel), Adrienn Tothpal and Eszter Kovacs (Semmelweis University, Hungary [28, 29]) and Debby Bogaert and Anne Wyllie (UMC, Utrecht [30]) (Table 1). Capsule-switch variants generated on the TIGR4 genetic background and the serotype 6B knockout strain were provided by Marc Lipsitch and generated as previously described [31]. Additional capsule-knockout strains were generated by replacement of the capsule biosynthesis locus with the Sweet Janus cassette [32].

**Table 1:** Pneumococcal strains used in this study

Source of isolate	Number of isolates	Country	Serotypes
Invasive pneumococcal disease (sepsis, meningitis)	40	US (CDC)	1, 2, 3, 4, 6A, 6B, 6C, 6D, 7C, 7F, 8, 9 N, 9 V, 10A, 10F, 11A, 11B, 12F, 13, 14, 15A, 15B, 15C, 15F, 17F, 18A, 18B, 18C, 19A, 19B, 20, 22F, 23F, 29, 31, 33F, 34, 35A, 35B, 37
	2	Hungary	3, 6B/D
Pneumonia	8	Hungary	3, 8, 10A, 15A, 19F, 35B, 43/45/38, NT
Conjunctivitis	16		3, 21, 31, 34, 42, 11A, 15A, 15B, 16F, 19A, 19F, 23A, NT
Carriage	52	Israel	6B, 14, 15B/C, 19A, 19F, 23F, NT
	87	Hungary	1, 3, 8, 21, 31, 34, 38, 6A, 9 V, 10A, 11A, 15A, 15B/C, 16F, 18C, 19A, 19F, 22A, 22F, 23A, 23B, 23F, 24F, 28F, 35F
	51	Netherlands	3, 27, 10A, 11A, 15B/C, 16F, 19A, 19F, 22A, 22F, 23B, 33F, 35B, 35F, NT
Laboratory-generated genetic variants	4	Various	TIGR4 (cps-, 5, 14, 19F), 603 (6B, cps-), CDC-10A (10A, cps-), CDC-15B (15B, cps-)

The multi-locus sequence type was inferred for a subset of the isolates. These strains were subjected to Illumina NovaSeq sequencing [33], and MLST was determined using SRST2 [34]. This Whole Genome Shotgun project has been deposited at DDBJ/ENA/GenBank.

under the accessions WNHG00000000-WNIC00000000 (BioProject PRJNA590574, BioSample SAMN13335401-SAMN13335575).

#### Culture media

Pneumococcal isolates were stored at − 80 °C on Cryobeads (Cryobank, Copan Diagnostics, Murrieta, CA). Strains were routinely grown at 37 °C and 5% CO_2_ overnight on tryptic soy agar plates supplemented with 5% sheep blood (TSAII) (Thermo Fisher Scientific). Growth in broth culture was performed in BHI (Becton, Dickinson, and Co., Sparks, MD) with and without catalase (5000 units, Worthington Biochemical Corporation, Lakewood, NJ) for aerobic cultivation and with Oxyrase® (Oxyrase, Inc., West Mansfield, OH) diluted 1:10 to create an anaerobic environment.

### Growth experiment

Strains were streaked onto TSAII plates and incubated at 37 °C in a 5% CO2-enriched atmosphere overnight, then harvested into PBS to OD600 0.2 and diluted (6 fold) in BHI with or without catalase or Oxyrase. Growth was monitored in sterile flat-bottomed 96-well microtiter plates (BRAND GMBH, Wertheim, Germany) for 24 h in a microplate reader (BioTek ELx808) with a built-in incubator, reading the optical density at 600 nm every 30 min, after 5 s shaking (Gen5 program). Each strain was tested in all three oxygen conditions and across the full range of temperatures (30–39 °C). An anaerobic control strain (*Bacteroides thetaiotaomicron*) was used to confirm the elimination of oxygen by Oxyrase.

### Data analysis

Each growth curve was blanked by subtracting the OD600 reading at t = 0 for that well. In instances where the t = 30 min measurement was lower than the t = 0 measurement due to measurement error at the first time point, the OD600 at t = 30 m was subtracted instead.

We extracted three characteristics from each of the growth curves: maximum density achieved, length of the lag phase, and density achieved at an early time point. The length of the lag phase was determined by fitting a smoothing spline to the log-transformed growth curves (smooth.spline function in R, with a smoothing parameter of 0.5) and calculating the second derivative of this curve. The maximum of the second derivative gives the point at which the growth rate increases the most, corresponding to the transition from stationary phase to log phase. We were also interested in evaluating the density at an early time point. However, what constitutes an ‘early’ time point likely varies by environmental conditions and source of the isolates. Therefore, we determined the mean time when log-phase growth began for each temperature/oxygen/site of isolate combination, and then determined the OD600 for the corresponding isolates 1 h after this point.

To quantify variations in the growth characteristics by serotype, site of isolation, and environmental condition while adjusting for repeated measurements and strain-to-strain variations, we used linear mixed effects models (lme4 package in R) [35]. The outcome variable was maximum OD600, fixed effects variables included serotype, temperature (categorical), oxygen (aerobic+catalase, aerobic without catalase, anaerobic), site of isolation (categorical), and a random intercept for each isolate. Certain interactions among the fixed effects were also evaluated to test specific hypotheses (site of isolate*oxygen; oxygen*temperature; serotype*oxygen). The significance of these interactions at different levels was evaluated using the interactionMeans function in the phia package in R [36].

## Supplementary information

**Additional file 1:** **Figure S1.** Isolate-specific random effects, organized by serotype. The random effect indicates how far above or below a strain is in maximum density achieved, lag length, growth rate, or density at an early time point. Serotype is not adjusted for in this model. The random effects are effectively an average residual for the isolate, after adjusting for temperature, site of isolation, and presence of oxygen. Each dot represents an individual isolate.

**Additional file 2:** **Figure S2.** Isolate-specific random effects, organized by serotypes and genetic lineage. The random effect indicates how far above or below a strain is in maximum density achieved, on average, after adjusting for temperature, site of isolation, and presence of oxygen. Serotype is not adjusted for in this model. Each dot represents an individual isolate; within a serotype dots of the same shape share an MLST type. Only isolates for which we have sequence data are included in the plot.

**Additional file 3:** **Figure S3.** Growth curves for capsule-switch variants on the TIGR4 background and capsule knockout strains.

**Additional file 4:** **Figure S4.** Growth curves for wild type and capsule knockout of a serotype 15B strain.

**Additional file 5: Figure S5.** Growth curves for wild type and capsule knockout of a serotype 10A strain.

**Additional file 6:** **Figure S6.** Growth curves for wild type and capsule knockout of a serotype 6B strain.

## Data Availability

The raw data and an R Markdown file are available in a github repository (https://github.com/weinbergerlab/GrowthVariation) and can be used to fully re-create the analyses presented here.

## Abbreviation

IPD: Invasive pneumococcal disease
