# Supplementary figures and images for: Variation of growth characteristics of pneumococcus with environmental conditions
Source: BMC Microbiol. 2019 Dec 26;19:304. doi: 10.1186/s12866-019-1671-8 (PMC6933730; doi:10.1186/s12866-019-1671-8)

Figure S1

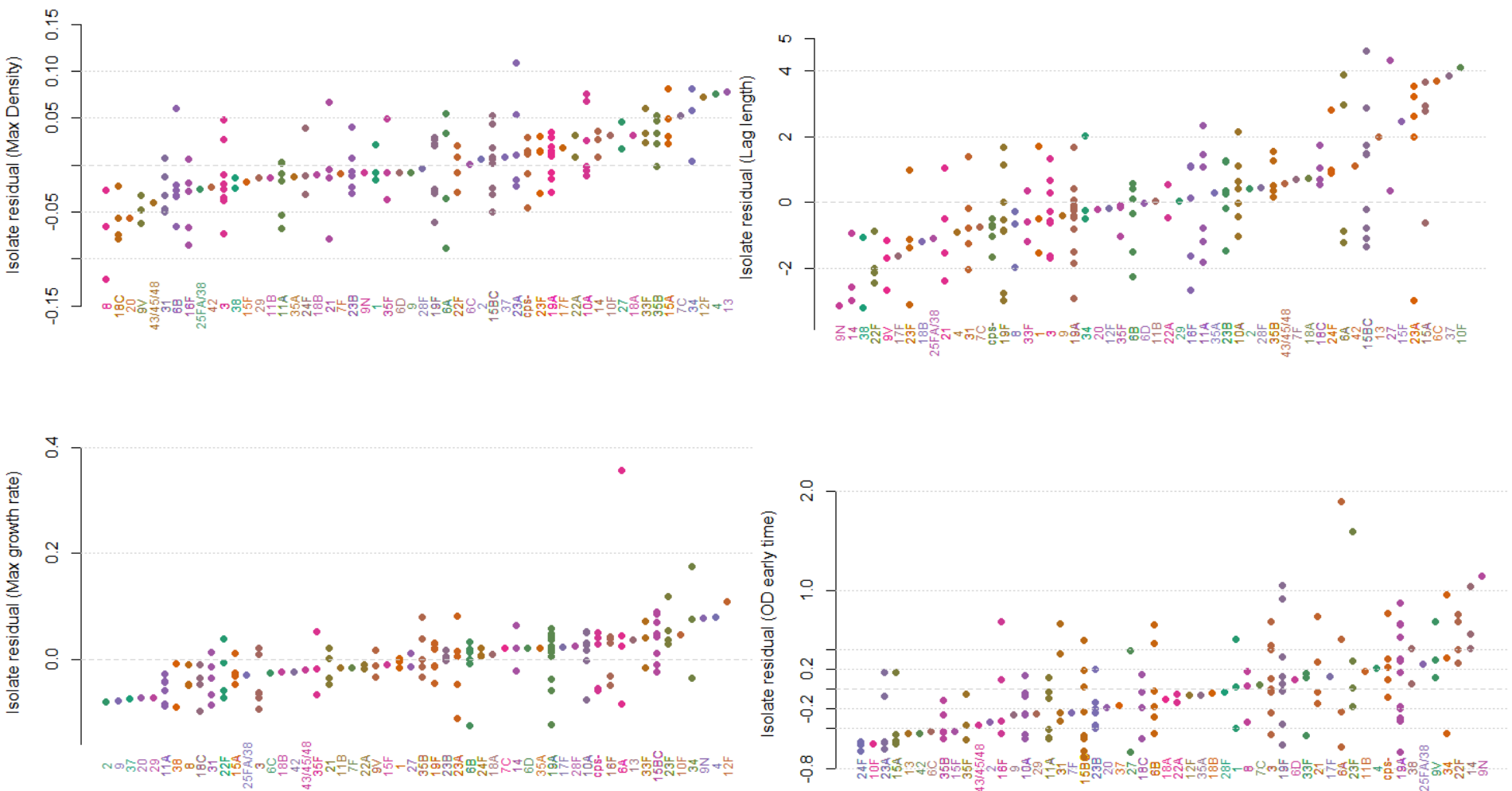

Supplement: Supplementary file 1 — Additional file 1: Figure S1. Isolate-specific random effects, organized by serotype. The random effect indicates how far above or below a strain is in maximum density achieved, lag length, growth rate, or density at an early time point. Serotype is not adjusted for in this model. The random effects are effectively an average residual for the isolate, after adjusting for temperature, site of isolation, and presence of oxygen. Each dot represents an individual isolate. [file 12866_2019_1671_MOESM1_ESM.pdf]

Figure S2

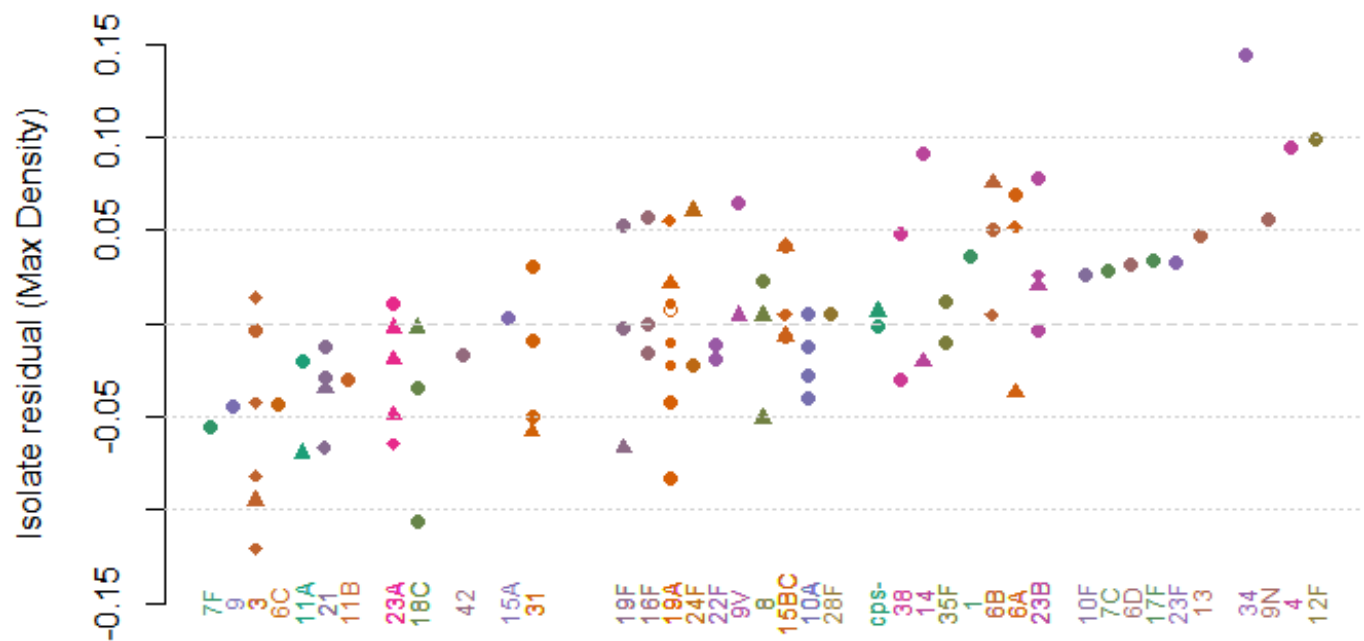

Supplement: Supplementary file 2 — Additional file 2: Figure S2. Isolate-specific random effects, organized by serotypes and genetic lineage. The random effect indicates how far above or below a strain is in maximum density achieved, on average, after adjusting for temperature, site of isolation, and presence of oxygen. Serotype is not adjusted for in this model. Each dot represents an individual isolate; within a serotype dots of the same shape share an MLST type. Only isolates for which we have sequence data are included in the plot. [file 12866_2019_1671_MOESM2_ESM.pdf]

Figure S3

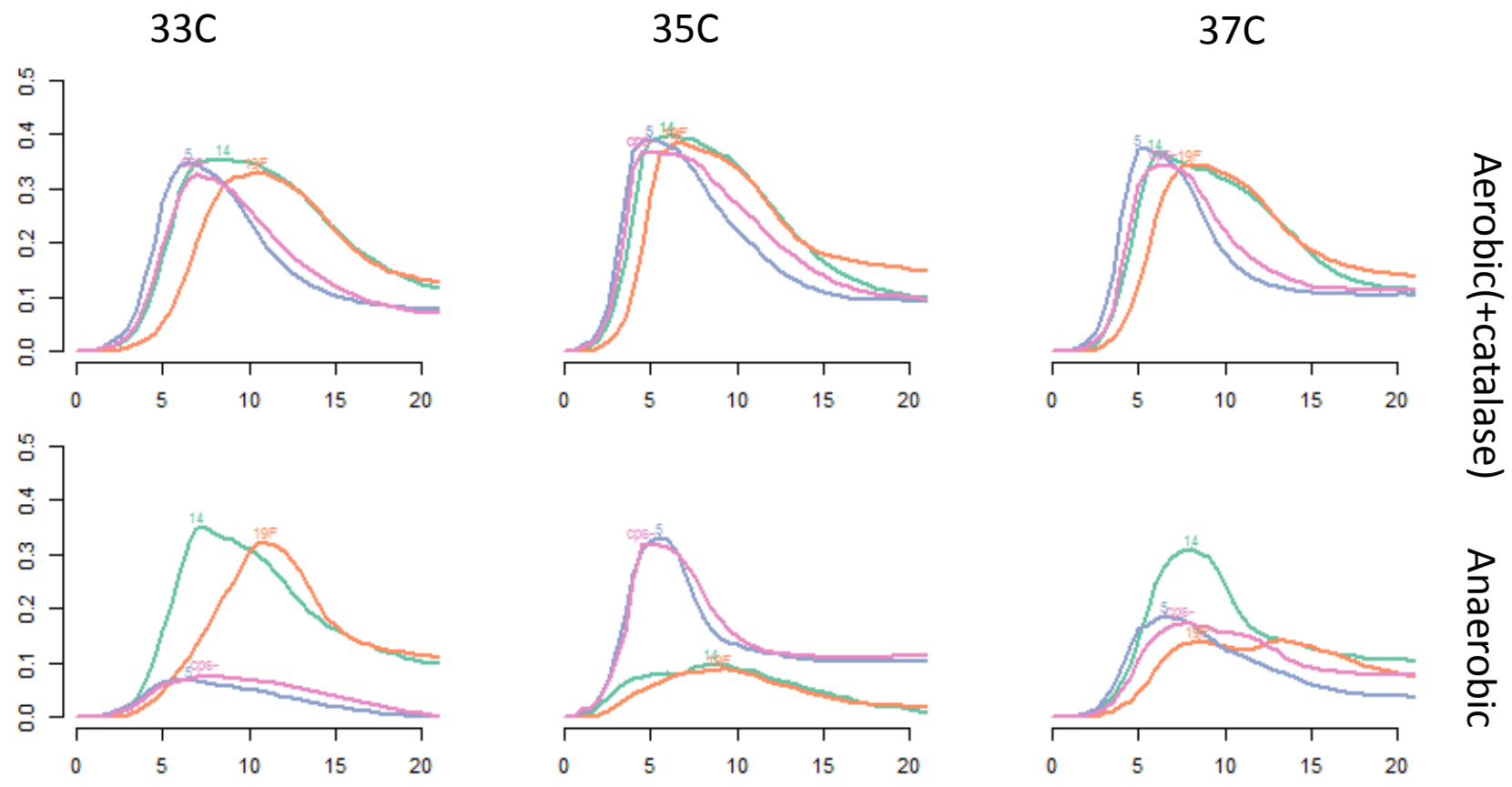

Supplement: Supplementary file 3 — Additional file 3: Figure S3. Growth curves for capsule-switch variants on the TIGR4 background and capsule knockout strains. [file 12866_2019_1671_MOESM3_ESM.pdf]

Figure S4

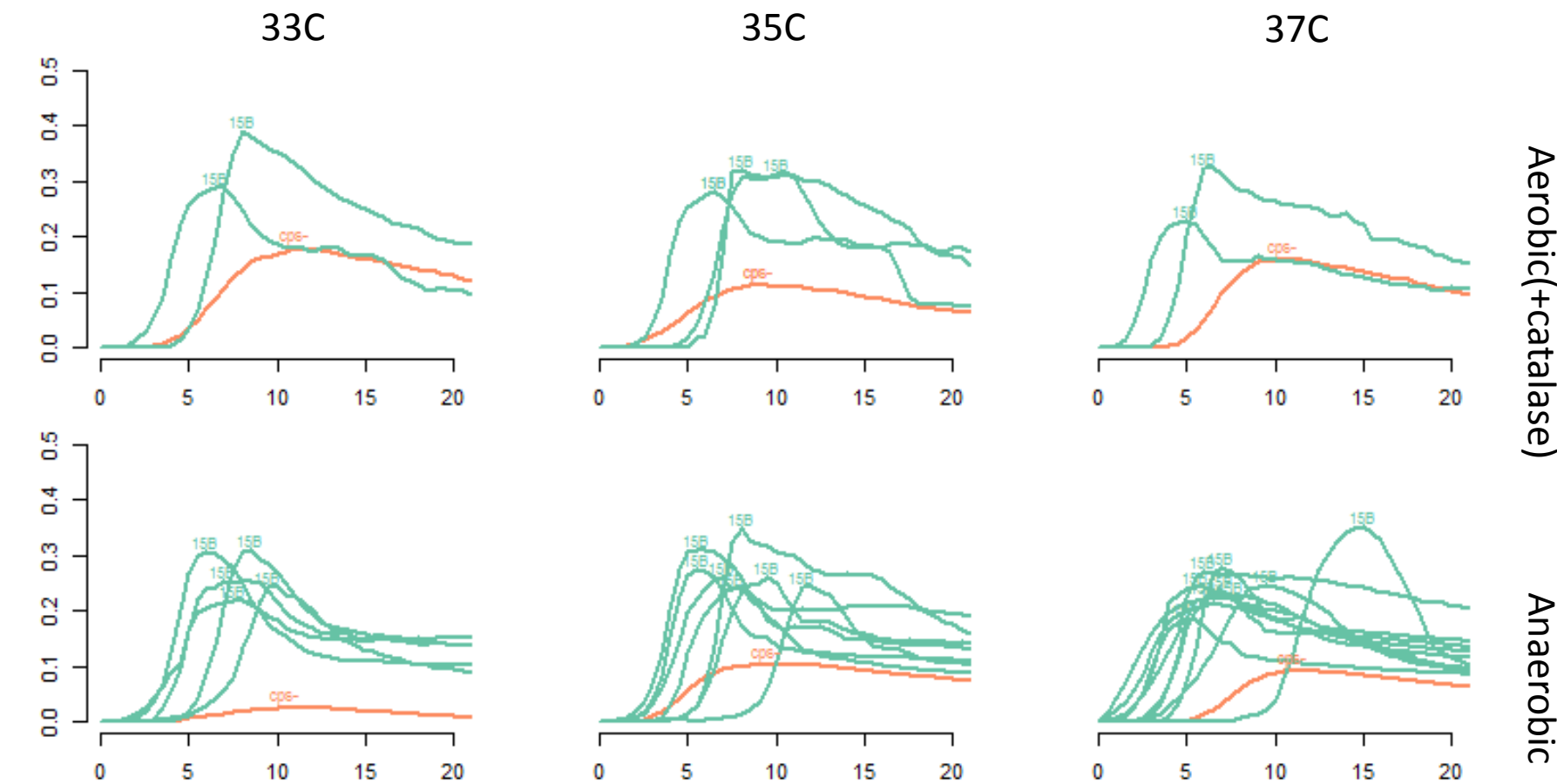

Supplement: Supplementary file 4 — Additional file 4: Figure S4. Growth curves for wild type and capsule knockout of a serotype 15B strain. [file 12866_2019_1671_MOESM4_ESM.pdf]

Figure S5

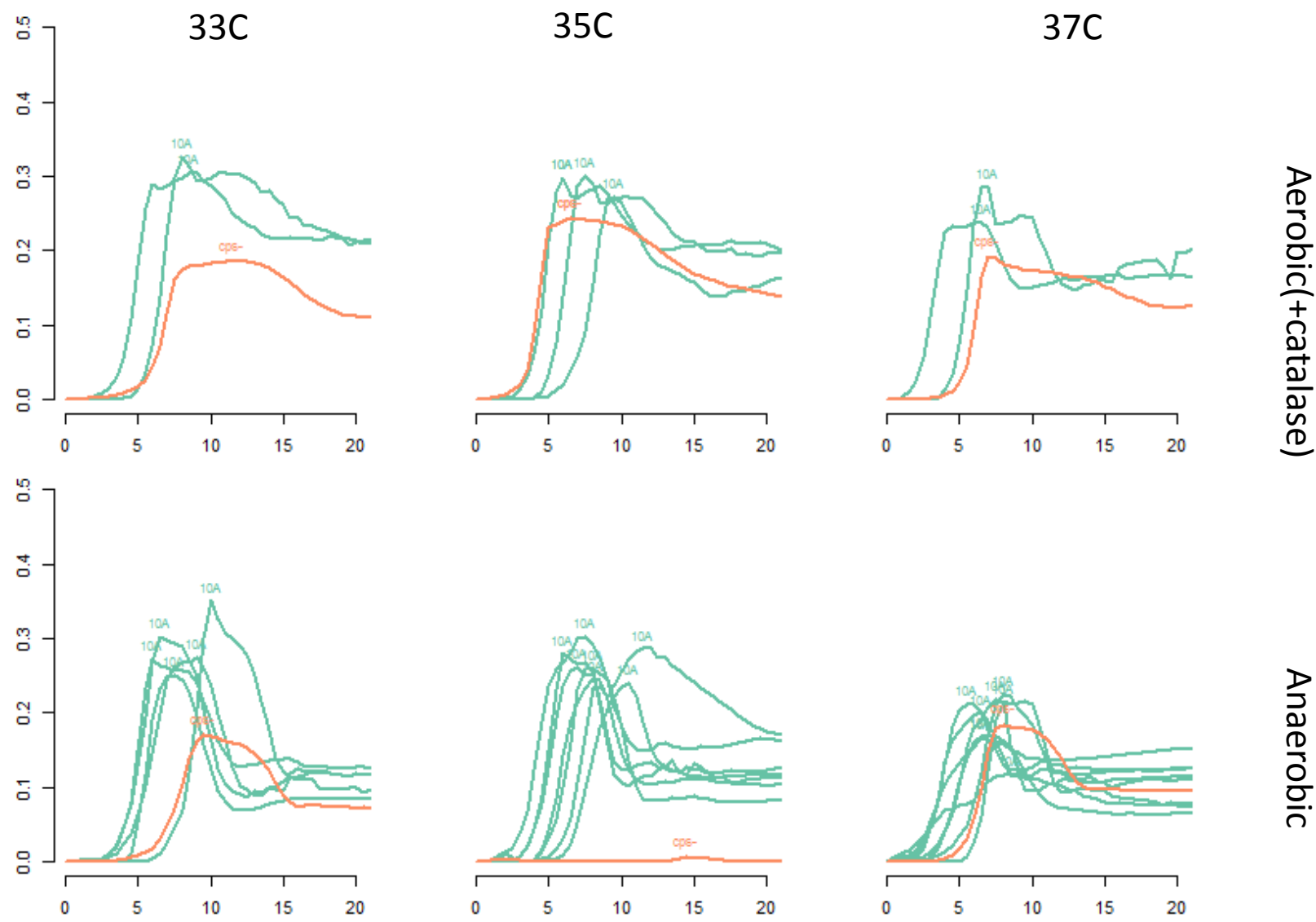

Supplement: Supplementary file 5 — Additional file 5: Figure S5. Growth curves for wild type and capsule knockout of a serotype 10A strain. [file 12866_2019_1671_MOESM5_ESM.pdf]

Figure S6

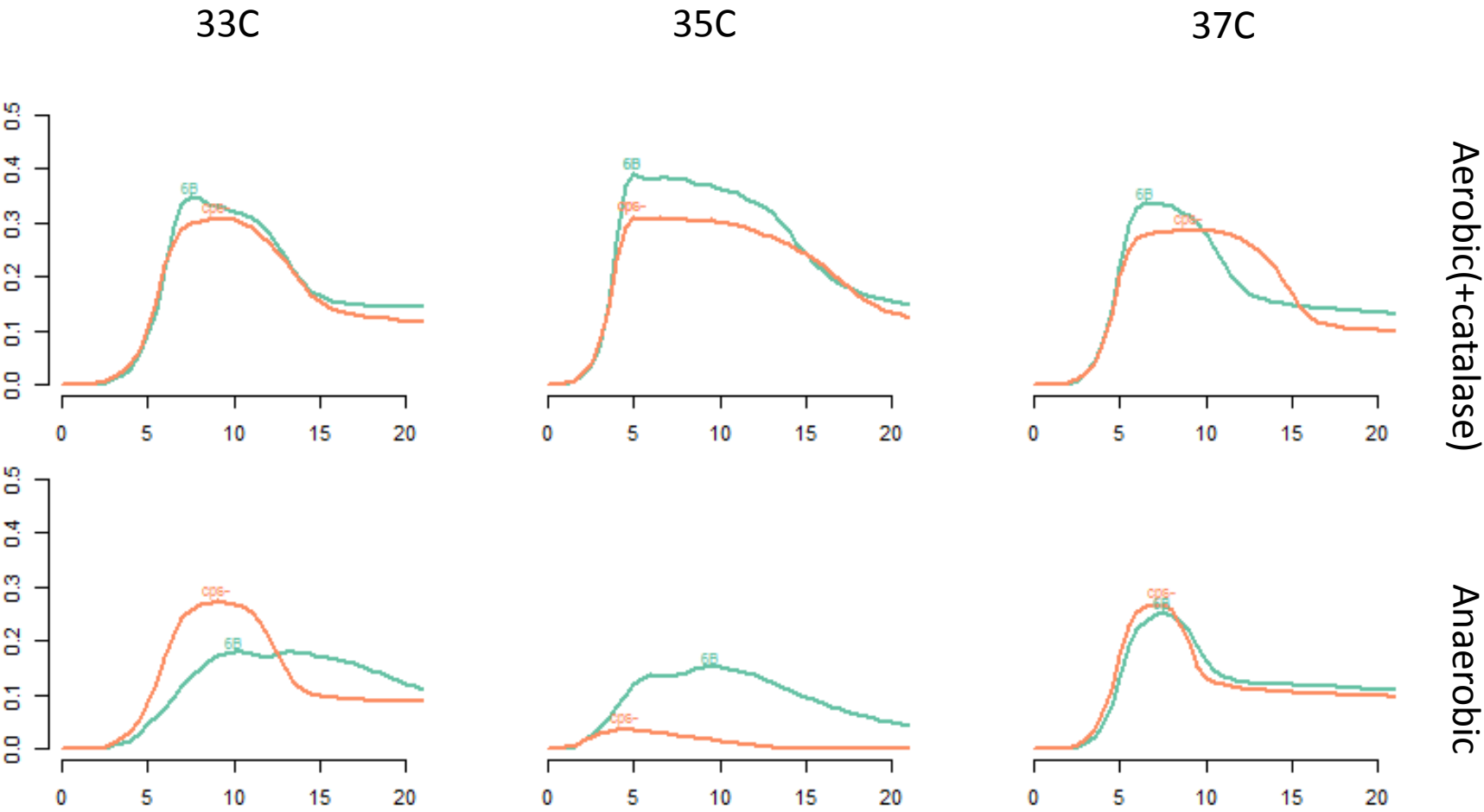

Supplement: Supplementary file 6 — Additional file 6: Figure S6. Growth curves for wild type and capsule knockout of a serotype 6B strain. [file 12866_2019_1671_MOESM6_ESM.pdf]
